# Feasibility of diagnosing major depressive disorder with a panel of serum and urine biomarkers

**DOI:** 10.1192/bjo.2026.11044

**Published:** 2026-06-15

**Authors:** Mike C. Jentsch, Huibert Burger, Marjolein B. M. Meddens, Sjoerd M. van Belkum, Brenda W. J. H. Penninx, Marcus J. M. Meddens, Robert A. Schoevers

**Affiliations:** Department of Psychiatry, University Medical Center Groningen, Groningen, The Netherlands; Department of General Practice, University Medical Center Groningen, Groningen, The Netherlands; Department of Radiology and Nuclear Medicine, University Medical Center Utrecht, Utrecht, The Netherlands; Department of Psychiatry, Amsterdam University Medical Center, Amsterdam, The Netherlands; Independent Researcher, Deventer, The Netherlands

**Keywords:** Major depressive disorder, TRIPOD, biomarker panel, machine learning, quantile-based prediction

## Abstract

**Background:**

Various biomarkers have been identified as being associated with the pathophysiology of major depression, with the potential to be utilised within an objective laboratory test for the diagnosis of depression, based on machine learning techniques.

**Aims:**

This study aims to build on previous results by modelling, in a larger and more heterogeneous cohort, the joint diagnostic accuracy of urine and serum-based biomarkers that showed predictive value for depression in our previous work.

**Method:**

A novel, multivariable, machine learning-based diagnostic tool for depression was tested on a combination of 34 urine- and serum-based biomarkers among 160 people diagnosed with major depressive disorder (MDD) and 120 controls, split into 3 different cohorts. Quantile-based prediction was applied to construct a biomarker-based diagnostic model (BDM) yielding a score for each biomarker. The sum score for each participant was used to calculate an area under the receiver operating characteristic curve (AUC) as a measure of discriminatory power.

**Results:**

We demonstrated that the BDM after internal validation had good discriminatory power, with an AUC of 0.81. Further internal–external validation by calculating individual depression probability scores for each separate cohort resulted in an AUC of between 0.62 and 0.72.

**Conclusions:**

In terms of clinical applicability, the present study shows that the combination of biomarkers and a machine learning model can discriminate between MDD and healthy controls with a modest level of diagnostic accuracy. A biomarker test could have potential added value for the future diagnostic toolkit, but this does require further research.

Major depressive disorder (MDD) is one of the most prominent causes of disability worldwide, with an estimated lifetime prevalence of approximately 15%.^1^ Currently, MDD is a syndromal diagnosis based on a combination of mostly subjectively experienced symptoms and functional impairment, documented using (semi-)structured clinical interviews combined with clinical assessments and categorised according to the ICD or DSM.^2–4^

Symptoms of MDD have considerable overlap with other psychiatric and physical disorders, making an accurate differential diagnosis challenging. However, a timely diagnosis of MDD is key, as delay means it takes longer to receive adequate treatment, a worse prognosis, increased patient burden and higher healthcare costs.^5–7^

In countries with a healthcare system in which general practitioners (GPs) and their mental health nurses are gatekeepers, they are responsible for recognising and diagnosing MDD and first treatment steps, and, if not successful, for referring patients to specialty care. Although the overall specificity of a GP diagnosis seems fair, with a meta-analytic specificity of 81.3%, only around half of patients with depression are recognised by their GP (weighted sensitivity 50.1%). Another meta-analytic study showed similar specificity, albeit with a lower sensitivity (36.4%).^8^ This means that a considerable amount of patients with MDD fail to receive early treatment because they are not recognised as having MDD in primary care.

Although it has been shown that the use of self-report questionnaires and enhancing GPs’ confidence in the ability to detect depressive symptoms can improve MDD recognition,^9,10^ it is likely that cases will still be missed because of the heterogenous nature of MDD.^11^ It has been proposed that adding an objective laboratory test based on biomarkers of the underlying biological pathways involved in MDD to the total diagnostic arsenal may enhance its recognition.^12–14^ Single biomarkers are not suitable for diagnosis because they are involved in various psychiatric disorders.^12^ Therefore, efforts have been made to combine biomarkers, thereby increasing discriminatory power and diagnostic value.^15,16^ Still, even when biomarkers are combined into a panel, limitations in terms of distribution of biomarkers within healthy and diseased cases, as well as individual patients’ characteristics, may influence the accuracy of biomarker-based diagnostic tests.^17–19^ Previously, we demonstrated that by using a statistical method that uses tails rather than means of predictor distributions and a specific set of biomarkers covering the major pathophysiological hypotheses of MDD, we were able to create a depression probability score that accurately discriminated between patients with MDD and healthy persons,^19^ by measuring a combination of 21 serum and 19 urine biomarkers within 40 MDD versus 40 control participants; we also showed within another study that the discriminatory power of the score improved when development was stratified by gender.^17^

In the present study, we build on our previous results by modelling, in a larger and more heterogeneous cohort, the joint diagnostic accuracy of urine- and serum-based biomarkers that showed predictive value for depression in our previous work. Specifically, we use machine learning to develop and internally and internally–externally validate a multivariable biomarker-based diagnostic model (BDM) that may be used to calculate individual depression probability scores.

## Method

### Study population

The study population included participants from the Mood Treatment with Antidepressents or Running (MOTAR),^20,21^ targeted Pulsed ElectroMagnetic Fields (tPEMF)^22^ and Peaks in the Delta East Netherlands/Vilnius (PIDON/Vilnius cohorts),^19^ comprising 160 cases of MDD and 120 controls. The authors assert that all procedures contributing to this work comply with the ethical standards of the relevant national and institutional committees on human experimentation and with the Helsinki Declaration of 1975, as revised in 2013. All procedures involving human patients were approved by their respective medical ethics boards. The PIDON/Vilnius study was approved by the Medical Ethical Committee of the Isala Clinics, Zwolle, The Netherlands (registration number 11.0563, 11 November 2011). The tPEMF study was approved by the Medical Ethical Committee of the University Medical Center Groningen, Groningen, The Netherlands (registration number 2012.039, 16 April 2012). The MOTAR study was approved by the Medical Ethics Committee of VU Medical Center, Amsterdam, The Netherlands (registration number 2012–064, 23 May 2012). Written informed consent was obtained from all participants. Participants were selected based on MDD status and completeness of data (age, gender and MDD status); for the PIDON/Vilnius study, it was required that the data were not previously used within the study by van Buel et al, which used a subset of the cohort for the analysis.^19^ Important characteristics of each cohort are depicted in Table 1. Controls for the current analysis were only from the MOTAR and PIDON cohorts because no healthy controls were included within the tPEMF study. Ethnicity was recorded using the different intake questionnaires administered in the various studies, according to the procedures described in the associated publications.

**Table 1:** Overview of individual cohorts comprising the total study population Table 1 long description.

Key study characteristics								
Cohort	Total size	Participant status		Study design	Recruitment	MDD diagnosis	Inclusion criteria	Exclusion criteria
		MDD	Control					
MOTAR	152	83	69	16 weeks, two-arm treatment intervention study, partially randomised	Participants were recruited between 2012 and 2019 at a mental healthcare institution (GGZ InGeest).	Participants were diagnosed at baseline using the DSM-IV and Composite International Diagnostic Interview. Inclusion criteria cases: having a current MDD diagnosis and aged between 18 and 70 years.	No lifetime MDD or anxiety disorder	Antidepressant use in the past 2 weeks, current use of other psychotropic drugs (except for benzodiazepines), other clinically severe psychiatric disorders
PIDON/Vilnius	84	27	57	Cross-sectional cohort study	Participants were recruited in collaboration with general practitioners, psychiatric care organisations and through advertisements in local and national newspapers. Participants were recruited from The Netherlands and Vilnius.	MINI diagnostic interview, age 18–65 years, a HRSD-17 score equal to or higher than 11	No lifetime major depressive or anxiety disorder	Pregnancy, presence of confounding psychiatric disorders
tPEMF	50	50	0	Sham-controlled double-blind multicentre trial	Participants were recruited from major mental healthcare institutions from the northern part of The Netherlands, with MDD based on the DSM-IV criteria for MDD, who were in a first or recurrent depressive episode as assessed by the MINI.	HRSD-17	Presence of at least a moderately severe depression (>17 on HRSD-17), non-responsiveness to one or more antidepressants given for at least 4 weeks during the current episode, age between 18 and 80 years	MDD with psychotic features, other major psychiatric disorders, neurological disorders, suicidal thoughts, alcohol or drug misuse, changes in medication treatment scheme during the course of the study, use of benzodiazepines

MDD, major depressive disorder; MOTAR, Mood Treatment with Antidepressents or Running; GGZ InGeest, Geestelijke (mental) Gezondheidszorg (healthcare); PIDON, Peaks in the Delta East Netherlands; MINI, Mini International Neuropsychiatric Interview; HRSD-17, 17-item Hamilton Rating Scale for Depression; tPEMF, targeted Pulsed ElectroMagnetic Fields.

### Blood and urine sampling

Blood samples were collected from all cohorts by venipuncture, allowed to clot, centrifuged, aliquoted and subsequently stored at −80°C. Early morning (PIDON, Vilnius and tPEMF) or 24 h (MOTAR) urine samples were collected, centrifuged, aliquoted and stored at −20°C. Blood and urine centrifuge protocols for the tPEMF and MOTAR samples included centrifuging blood for 10 min at 1300 *g,* whereas urine samples were centrifuged for 10 min at 2500 *g*. For the PIDON and Vilnius samples, blood was centrifuged for 10 min at 3000 *g,* and early morning urine was centrifuged for 10 min at 1000 *g*.

### Biomarkers panel and measurements

Biomarker selection was based on previous studies.^17,19^ From these studies, only biomarkers that showed a high diagnostic value for MDD were selected. These biomarkers were supplemented with acetyl L-carnitine because recent literature has indicated that this biomarker may be implicated in the pathophysiology of MDD.^23^ Supplementary File 1 available at https://doi.org/10.1192/bjo.2026.11044 provides an overview of the biomarkers assessed within this study. The biomarker panel comprised 19 serum biomarkers and 15 urine biomarkers. To adjust for renal function, urine biomarker levels were expressed as the ratio of the concentration of biomarker relative to the concentration of creatinine.

Individual biomarker levels within serum and urine samples were determined using enzyme-linked immunosorbent assay (ELISA). The different ELISAs were purchased from various vendors (see Supplementary File 1). ELISA procedures were performed using specific standard operating procedures, in which all experimental variables are recorded for full experimental traceability. Standard operating procedures were based on manufacturer’s instructions, with the following adjustments: addition of calibrators, extra dilution steps and adjustments of pipetting volumes to a minimum of 20 µl to reduce pipetting errors during dilution steps. An ELISA plate washer (Bio-Rad PW40) was used for all washing steps. TMB absorption measurements were performed on a microtitre plate reader (Thermo Multiskan Spectrum) at 450 nm, using 620 nm as a reference wavelength. Unknown biomarker concentrations were determined using a four-parameter logistic regression fit without weight factors of the calibration standard. A sample handling management system with two-dimensional barcoded tubes (Micronics) for sample identification was used. Samples were randomly positioned on the seven ELISA plates. Randomisation was carried out for each biomarker/body fluid combination. Each biomarker was assessed in one batch, to control for variability of the research ELISA kits. Each ELISA plate contained quality control samples with known concentrations within the low and high range. Quality control samples were prepared by pooling two to four unused samples that were tested to be within the desired range. Quality control samples were aliquoted in two-dimensional Micronics tubes, and on each ELISA plate, four quality control samples were added (two low, two high). The position on the ELISA plate was randomly assigned to prevent position bias. An additional performance indicator called the relative variability index (RVI) was developed and used to control for assay versus biological variability. The full description for calculating the RVI can be found within Supplementary File 2.

### Data analytic strategy

The data analytic strategy was developed in accordance with the Transparent Reporting of a multivariable prediction model for Individual Prognosis Or Diagnosis + Artificial Intelligence (TRIPOD + AI checklist),^24^ and consisted of three overarching steps. The first step (step A) involved characterising the study population including biomarker levels according to MDD status, and assessment of assay performance (RVI). In the following two steps (B and C), we conducted a ‘type C’ analysis as defined by Collins et al,^25^ involving (step B) model development using all available data from the combined cohorts, followed by (step C) performance evaluation via internal–external cross-validation with cohort as the splitting unit. Additionally, we implemented two supplementary procedures: (a) an internal validation using *k*-fold cross-validation to assess potential overfitting, and (b) the assessment of potential gender interaction by using a logistic regression model. Both supplementary analyses were based on the model developed using data from the combined cohorts.

### Step A

Descriptive statistics were calculated for demographic characteristics of the total study population (gender, ethnicity, age) according to MDD status, by using the Fisher exact test for categorical variables and *t*-test for continuous variables. Differences in mean biomarker levels between healthy controls and cases of MDD were assessed using one-way analysis of variance. Levene’s test was used to assess variance differences for each biomarker. Assay performance was assessed by calculating the RVI (in line with the method described in Supplementary File 2).

### Step B

**Fig. 1:**
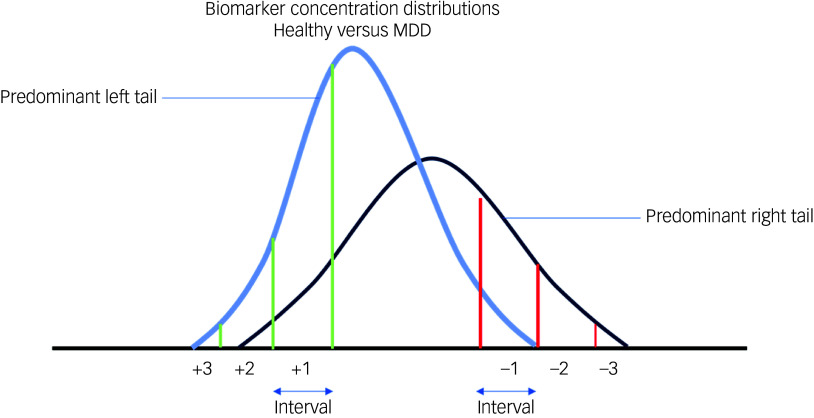
Quantile-based prediction model development based on biomarker concentration distributions health (black line) versus disease (blue line). Determining the total BDM score: biomarker concentrations falling within the red line areas receive a score of −1, −2 or −3 (associated with healthy). Biomarker concentrations falling within the green line areas receive a score of +1, +2 or +3 (associated with disease). Biomarker concentrations falling within the area between the green and red line receive a score of 0 (associated with not contributing to disease or healthy status). The sum of all scores for healthy and disease, the total BDM score, is used to construct a receiver operating characteristic for which an area under the curve can be calculated as a measure of discriminative power. BDM, biomarker-based diagnostic model; MDD, major depressive disorder.

Step B consisted of model development and refinement The diagnostic model included biomarkers that were identified as being potential predictors based on the statistical significance levels of the analyses of variance. The BDM was subsequently derived by using a machine learning technique, i.e. quantile-based prediction (QBP), which was developed for binary disease classification.^19,26^ In short, this machine learning method does not use mean differences in predictor values, but rather QBP assigns scores to certain percentiles in the left and right tails of empirical biomarker distributions in which a shift case versus control is observed. Based on which population (case or control) dominates a tail, either a positive or negative score of 1, 2 or 3 is assigned to percentiles of the biomarker distributions in the tail. The further out the percentile, the higher the ‘relative weight’ of the biomarker in predicting MDD, and thus the higher the absolute value of the score. Non-relevant biomarkers with similar distribution curves are omitted from the model and are automatically assigned a score of 0. The BDM total score is the sum of the biomarker specific scores. Figure 1 is a visual representation of how QBP is used in constructing the BDM and how the weight of a measurement is determined including model improvement by changing the limit values of the intervals used to assign a score based on biomarker concentration.^25^ The initial model development was performed on the total study cohort. The discriminative performance of the model was determined as the area under the curve (AUC) of the receiver operating characteristic (ROC) curve of the total BDM score. The statistical relevance of the AUCs was determined by permutation analysis.

After initial development, the model was refined through two procedures. The first procedure involved AUC optimisation of the sum of all scores by empirically loosening or strengthening the cut-off values for the percentiles used for determining the inclusion/exclusion of contributing biomarkers. Strengthening or loosening the cut-off criteria alters the relative weight put on a specific measurement, thereby increasing or decreasing the discriminative power of a single biomarker measurement. This is done by shifting the interval boundaries for assigning a biomarker-specific score, resulting in a reduction/increase of contributing biomarkers and an increase/decrease in the AUC. The second refinement procedure involved a further refinement of the model by selecting only predominant tails (tails associated with either health or disease) of individual biomarkers, meaning only selecting biomarkers associated with disease or health status. After each refinement procedure, the discriminative performance of the model was reassessed. This optimised model is further referred to as the final model. Based on this model we calculated sensitivity and specificity for several cut-offs.

### Step C

**Fig. 2:**
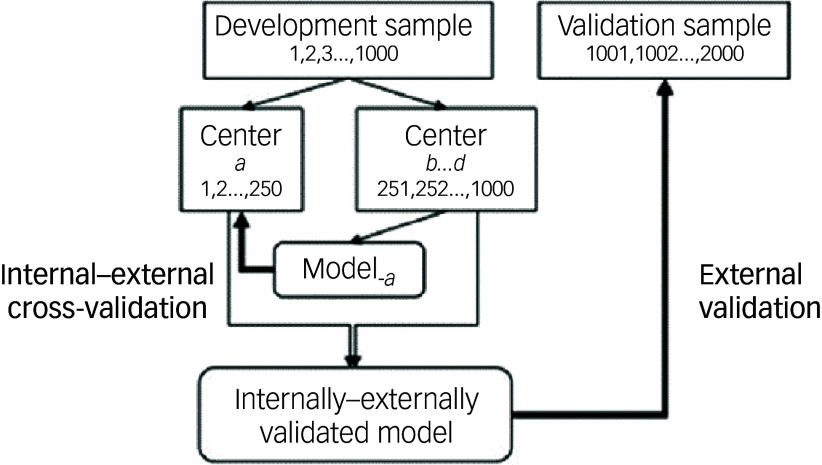
Figure is adapted with permission from Steyerberg and Harrel,^28^ and is a schematic representation of the internal–external cross validation applied within this study.

In step C, we integrally evaluated overfitting and variation in model performance by cohort. This was accomplished using an internal–external validation as presented within Fig. 2. Within the realm of individual patient data meta-analysis, internal–external cross-validation with study as the splitting unit has been employed to demonstrate the external validity of a predictive model.^27^ In short, two of the three individual cohorts were combined to serve as the training cohort, and the remaining cohort was used as the validation cohort. In the training cohort, the BDM was developed and refined just as in step B. This process was repeated three times in a rotating manner, each time leaving one cohort out to utilise as validation cohort. The total scores for each validation cohort were determined based on a score versus concentration table. This table was constructed based on the model developed using the training cohort. For each training cohort, the same control group was used (combination of controls from MOTAR and PIDON), which was re-used within the validation cohort in order to calculate a ROC for each validation cohort.

### Supplementary analyses

Because of the method of model development and refinement, the risk of overfitting was high. Therefore, to test for overfitting in the final model, a 10 × 5 fold cross-validation was performed as a way of internal validation using the biomarkers selected during the model refinement step. For each iteration, all participants were randomly assigned to one out of five test cohorts, keeping gender and disease status ratios equal between cohorts. For each test cohort, the training cohort consists of all participants not in the current testing cohort. The BDM was developed on the training cohorts calculating limits and scores in Microsoft Excel, and subsequently tested on the test cohort. This was repeated for all five test cohorts. The procedure was repeated ten times with different randomisations. For both the training and test cohorts, an average AUC is reported as the mean of all 50 (10 randomisations × 5 testing cohorts) realisations.

Gender interaction was assessed by testing a product term gender×BDM total score added to the independent variables comprising gender and BDM total score (main effects) in a logistic regression model, with MDD status as the dependent variable. This was done because gender may have a modifying effect on model performance, as evidenced by our previous work.^17^ For the final BDM, the total score was dichotomised and sensitivity and specificity were determined based on the Youden index. The Youden index calculates the performance of a diagnostic test.

The level of significance was set at 0.05, two-sided.

### Software packages used

Descriptive statistics of cohort composition were calculated in Microsoft Excel (mean, *t*-test), part of the Microsoft office 365 package, and Graphpad Prism 10 (https://www.graphpad.com) (Fisher exact test). Five-fold cross validation was performed using Microsoft Excel (template available upon request). Comparison of biomarker differences between cohorts and binary logistic regression analysis were performed using SPSS version 28 (IBM, Armonk, NY, USA; https://www.ibm.com/products/spss). Phenotype permutation was performed in LabVIEW (self developed tool based on LabVIEW platform available upon request). The internal/external validation was performed using both Microsoft Excel and SPSS version 28. For QBP analysis, calculation of ROC curves and the AUC was executed in LabVIEW, Microsoft Excel and/or SPSS version 28. Medcalc version 22 (MedCalc Software, Ostend, Belgium; https://www.medcalc.orgx) was used for determining sensitivity and specificity. All software programs were used on the Windows platform.

## Results

### Step A

#### Demographic characteristics and biomarker levels according to MDD status

Demographic characteristics of the study population are presented in Supplementary File 3. Ethnicity and gender showed no statistical relevant extremities. The mean age of the control group is slightly higher compared against the MDD (case) group (*P* = 0.027).

Differences in biomarker levels respective to MDD status are presented in Table 2. From the 34 biomarkers analysed, mean concentration of 13 biomarkers (7 in serum and 6 in urine) were significantly different between MDD and controls, and 11 biomarkers were significantly different based on variance. Mean differences in biomarkers in serum included calprotectin (higher levels versus control), cyclic adenosine monophosphate (cAMP) (lower levels versus control), acetyl-L-carnitine (higher levels versus control), thromboxane (lower levels versus control), leptin (higher levels versus control), myeloperoxidase (higher levels versus control) and total brain-derived neurotrophic factor (BDNF) (lower levels versus control). Within urine, significant biomarkers included acetyl-L-carnitine (higher levels versus control), substance P (lower levels versus control), herpes virus entry mediator (HVEM) (higher levels versus control), resistin (higher levels versus control), lipocalin 2 (higher levels versus control) and calprotectin (higher levels versus control). From these biomarkers, nine biomarkers (green colour in Table 2) actively contribute to the BDM model as developed in step B.

**Table 2:**
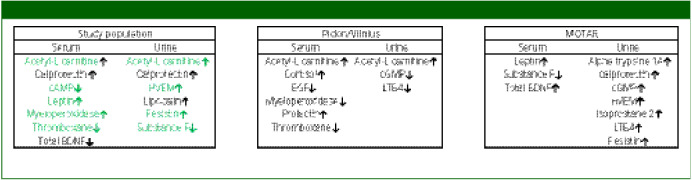
Significant biomarkers within the total study population and separate cohorts

cAMP, cyclic adenosine monophosphate; BDNF, brain-derived neurotrophic factor; HVEM, herpes virus entry mediator; PIDON, Peaks in the Delta East Netherlands; EGF, endothelial growth factor; cGMP, cyclic guanine monophosphate; LTB4, leukotriene B4; MOTAR, Mood Treatment with Antidepressants or Running; BD, biodepression. tPEMF is not depicted since no controls are used within the PEMF study. Supplementary File 9 shows the full measured mean concentrations of each group including SEM and *P* value. ↑ is indicating higher mean biomarker level in cases than in controls. ↓ is indicating lower mean biomarker level in cases than in controls. Green coloured biomarkers actively contribute to the development of the BD model.

#### Assay performance

To analyse the generated biomarker data and assure a high validity, an RVI factor was determined for each biomarker assay representing a biological performance indicator. The RVI was assessed in 34 assays. Of these, it appeared that four out of the 34 assays did not meet the criterion for acceptability. These were zonulin in serum (RVI = 2.7), alpha1-antitrypsin in serum (RVI = 2.1), BDNF in serum (RVI = 2.1) and acetyl-L-carnitine in serum (RVI = 2.7). All RVIs are presented within Supplementary File 2.

### Step B

#### Model development

Applying QBP to the data of the total population, including all 34 biomarkers, after empirically optimising the limits as part of step 1 refinement (16/10), resulted in the identification of 24 actively contributing biomarkers with an AUC of 0.82 (*P* < 0.001) after 2321 AUC permutations. Selecting predominant tails only, reduced the number of biomarkers to 19 and increased the AUC to 0.85.

**Fig. 3:**
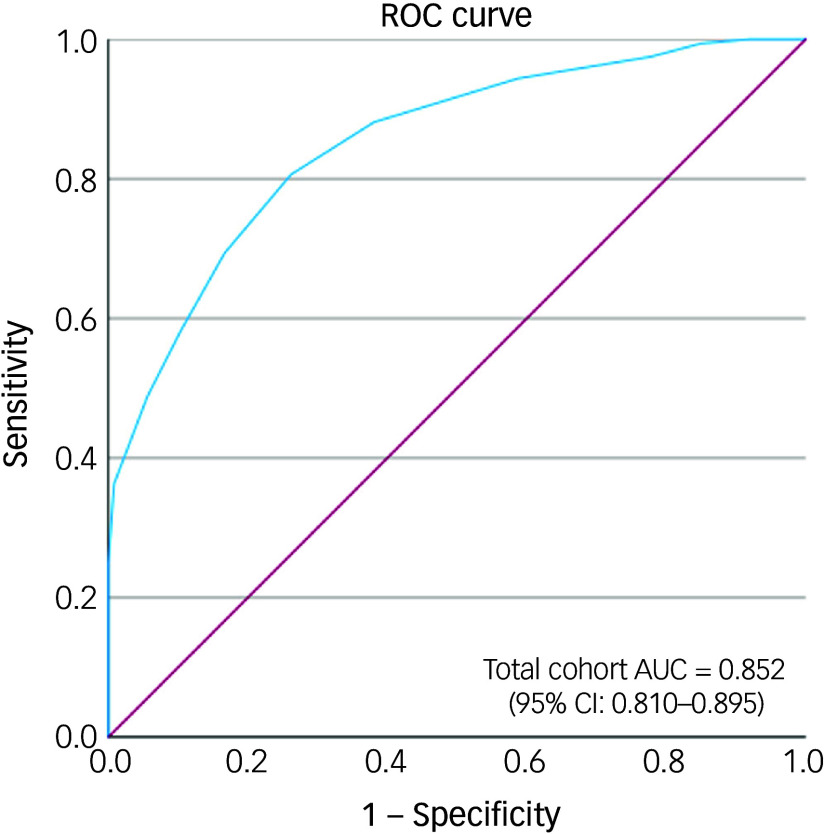
Receiver operating characteristic (ROC) curve of the bio-depression model score for the total study population obtained from the combined active serum and urine biomarkers determined with the quantile-based prediction and active tail-only selection. Area under the curve (AUC): 0.852 (95% CI: 0.810–0.895) (sensitivity: 80.6%, 95% CI: 73.64–86.44%; specificity: 73.8%, 95% CI: 65.23–81.24%) with 19 contributing biomarkers. Diagonal segments are produced by ties.

Biomarker performance of this model as assessed using ROC curve analysis is depicted in Fig. 3. It showed an AUC of 0.85 (95% CI: 0.81–0.9).

### Step C

#### Internal–external validation

As part of the internal–external validation of the BDM, a model was developed separately for each training cohort, which resulted in a consistent reduction of the number of contributing biomarkers to 13. From the 19 actively contributing biomarkers within the BDM developed on the total study population, 7 biomarkers actively contributed to the individual models within the individual *k* cohorts. Two biomarkers within urine – cGMP and LTB4 – only contribute to the BDM within the total study population. Limits used, AUC of the BDM and permutation analysis of the training cohorts are shown in Supplementary Table 4. Active biomarkers of the respective (training) cohorts are presented in Supplementary Table 5. Internal–external validation showed that based on the calculated depression probabilities scores, an AUC could be calculated for MOTAR of 0.623, for tPEMF of 0.670 and for PIDON/Vilnius of 0.721. The ROC curves for the internal–external validation of MOTAR, PIDON/Vilnius and tPEMF are shown in Supplementary File 6. BDM score versus concentration tables used for calculating the total score for the testing cohorts of the internal validation are presented in Supplementary File 7. Specificity and sensitivity of each individual cohort are presented in Supplementary File 8, including the calculated values for the BDM developed and refined on the study cohort as described in step B.

### Supplementary analyses

#### Internal validation

Applying a ten times five-fold cross validation of the final model on the study population resulted in a combined training AUC of 0.85 (specificity: 75%; sensitivity: 78%) and a combined testing AUC of 0.81 (specificity: 75%; sensitivity: 69%).

#### Gender interaction

Binary logistic regression showed that the BDM total score had an odds ratio of 1.60 (*P* < 0.001) (CI 1.37–1.86). No gender interaction was determined (odds ratio 1.2, CI 0.91–1.61; *P* = 0.2).

## Discussion

The current study investigated the joint diagnostic accuracy of a serum and urine biomarker-based diagnostic algorithm for the diagnosis of MDD within a large and heterogenous cohort of patients and controls. As a proof of concept, we created a score based on the final model that may be used to calculate individual depression probability scores. Our study thereby established directions for developing an effective, machine learning-based, multivariable, biomarker-based diagnostic tool for depression.

We have shown that our biomarker panel of 34 urine and serum combined biomarkers associated with MDD pathophysiology has the potential to discriminate between MDD and healthy participants by utilising a machine learning-based model. The potential overfitting and generalisability of the model were challenged by internal and internal–external validation methods, respectively. Although our internal validation procedure suggested moderate overfitting (decrease in AUC from 0.85 to 0.81), the internal–external cross-validation showed considerable performance heterogeneity across cohorts and limited generalisability (external validity), with substantially lower AUCs, ranging between 0.623 and 0.721. Therefore, current model performance would best be described as modest based upon our external validation.

The list of biomarkers potentially associated with MDD is large,^11,12,29^ with high variation in biomarker associations with MDD because of varying pathophysiology. This reality makes it hard to correctly identify suitable biomarkers for a diagnostic biomarker panel. To utilise the potential diagnostic information within these biomarkers, the use of machine learning can advance a potential promising biomarker panel to the next stage of development, as shown in two studies by Papakostas et al and Bilello et al.^15,16^

The strength of combining a selection of promising disease-associated biomarkers with a machine learning method was previously shown. In an earlier study, van Buel et al^19^ showed that with a combination of 17 biomarkers (8 urine and 9 serum biomarkers), a depression probability score could be generated that was able to accurately discriminate between patients with MDD and non-depressed individuals, with a ROC AUC of 0.96. These results were then used as the basis for our current study by including all relevant biomarkers from the study by van Buel et al and supplementing them with additional biomarkers based on new scientific insights. Five urine and six serum biomarkers from the study by van Buel et al also actively contributed to the AUC of 0.85 within the current study. Within urine, these biomarkers include cGMP, HVEM, isoprostane-2, substance P and thromboxane. Within serum, they include BDNF, cAMP, cortisol, leptin, thromboxane and TNF receptor 2. The AUC of 0.85 is lower than the AUC of 0.96, which was reported by van Buel et al, although the cross-validation AUCs show a similar pattern. A possible explanation of the lower AUC in our study compared with that in the study by van Buel et al is that, to the best of our knowledge, in the latter study no shrinkage or any other correction method for overfitting and optimism was applied during the modelling process, e.g. bootstrapping. An additional explanation is that the current cohort is more heterogenous because of the combination of several cohorts with an increased number of participants, likely representing a broader clinical and biological spectrum. Nevertheless, 11 biomarkers from the van Buel et al study contributed significantly to the performance within the current study, suggesting that these biomarkers cover the heterogenous nature of MDD and are therefore good candidates for further biomarker panel development toward diagnostical applications. Within the current developed model, gender shows no interaction effect, which is in line with what was previously reported by Jentsch et al.^17^ Developing the model specifically, i.e. stratified for gender, on the other hand, could improve the performance of the model by better taking gender-specific biomarkers into account, as was previously indicated. Recent insights showing various gender-specific differences within MDD pathophysiology^30^ support such a view. Therefore, model development specifically for gender deserves further investigation. Finally, fine-tuning the algorithm – potentially at the price of increased overfitting – also resulted in a better panel performance, as was evidenced by a higher AUC when selecting only active relevant tails instead of relevant biomarkers. This would indicate that the model performance better when noise is removed (by selecting only disease associated tails of biomarker distributions).

Although our results are promising, the present study also has limitations. First of all, we performed various types of validation procedures to test our panel for diagnostic potential. This showed that the diagnostic potential of the panel is there, but that it is sensitive to the specific composition of contributing biomarkers within a specific cohort, affecting the discriminative power. This was specifically evidenced by the internal–external validation showing a sharp reduction in discriminative power depending on cohort. This likely indicates that the composition and representativeness of a specific cohort plays an important role in the generalisability of biomarker panel performance to a wider population. Part of these differences could most likely also be accounted to differences in biomarker levels related to factors such as age, medication and ethnicity,^31–33^ which can affect the accuracy of the BDM as previously shown for gender, but also for ethnicity.^33^ These covariates were not incorporated into the model development with the exception of gender (because of the results reported in Jentsch et al^17^), as the aim of the current study was to develop a model based solely on biomarker differences associated with disease status. Therefore, future investigations should focus on these factors. Another factor that places somewhat of a limitation on the interpretation of our study is the use of one pooled group of controls utilised for developing and testing the BDM for each subcohort. By pooling the controls, the independence of cohorts is reduced. Furthermore, although apparently moderate according to our five-fold cross-validation, overfitting is likely to have played a considerable role in the BDM construction because of the amount of model improvement steps taken. Although most likely contributing to the overfitting, improvement steps are considered essential for BDM development and effectiveness because they provide a better selection of contributing biomarkers associated with disease and health. Another limitation is the use of a case–control design, which entails non-random sampling of the target population (i.e. patients suspected of having MDD), potentially leading to selection bias.^34^ As a result, a true external validation would include a cohort of patients suspected of MDD, avoiding a case–control approach, and ensuring that none of the data from this cohort contributed in any way to model development. In summary, these limitations most likely contributed to the concerns of generalisability as evidenced by the internal–external validation approach. Next to potential limitations in terms of biomarker panel validity, the current study also has some potential analytical limitations. The use of biochemistry approaches such as ELISA is advantageous because of its high sensitivity. The disadvantage of ELISA methods, however, is that they are not only prone to biological variations within samples, but also to variations in assay performance that can have an impact on correctly assessing and interpreting results. It is therefore imperative to implement a basic level of quality assurance into your assays to control for assay variations.^35^ Therefore, we implemented various quality assurance and assay performance parameters such as calculating the RVI for all assays. This showed that only for three assays the biological variation might be too high, potentially negatively affecting a correct assessment and interpretation of the results. Nevertheless, biomarkers with a low calculated RVI were not removed from this study, because they still contribute to model development. It is interesting to note that these biomarkers also did not actively contribute to the predictive model. Although basic quality assurance and assay performance parameters were used, it should be mentioned that all ELISAs are research and development kits, with only a basic level of determining assay performance parameters. Because of our use of the RVI and quality control samples and duplo measurements, the reliability of the generated results was positively influenced. This is also true for potential analytical variations introduced because of the slight differences in blood and urine sampling methods for the different cohorts. Potential variations introduced as a result of this are covered within the RVI calculation method. In terms of clinical applicability, we have shown that the developed biomarker panel consisting of various serum- and urine-based biomarkers is capable of discriminating between MDD and controls with considerable accuracy, exceeding the diagnostic accuracy of GPs based on current literature. However, to be relevant in clinical practice, a biomarker-based diagnostic test should ideally also demonstrate added value to additional assessments such as the depression screener Patient Health Questionnaire 9 (PHQ-9)^36^ (sensitivity: 85%; specificity: 89%^37^), even though such screeners are currently not widely implemented in clinical care^38^ A direct comparison between the BDM and the PHQ-9 could be valuable in future studies. Compared with our BDM, current literature shows AUC values for the PHQ-9 ranging between 0.77 and 0.973.^39,40^ In addition, a biological diagnostic tool might be considered of additional value in cases of diagnosing subtypes of depression, initiating the right treatment based on biological background or monitoring treatment response by monitoring biomarker changes associated with treatment response. To advance to such a stage of utilising a biological diagnostic tool, future research should focus first on performing a true external validation followed by freezing the acceptance criteria and the biomarkers included into the panel. Afterward, the panel can be developed further for depression subtyping and potentially treatment monitoring based on biological indicators in cooperation with current diagnostic tools.

In summary, the current study provides a good view of the feasibility of diagnosing MDD with a panel of serum and urine biomarkers. Machine learning approaches in combination with literature informed selection of biomarkers associated with disease are essential for achieving this. We have shown that utilising this approach results in a model with a good diagnostic accuracy even when subjected to internal external validation using heterogeneous patient data.

Before incorporating a model into routine practice, it is crucial to extensively externally validate and fine-tune it, e.g. by updating procedures. The model we presented here can therefore best be seen as an initial foundation for subsequent investigations. Subsequent research endeavours should include an external validation study, and ultimately an examination of the model’s impact and feasibility in practice. This may involve assessing factors like the ability of healthcare professionals to implement a biomarker-based model into practice, using a process evaluation.

## Supporting information

10.1192/bjo.2026.11044.sm001 Jentsch et al. supplementary material 1 Jentsch et al. supplementary material

10.1192/bjo.2026.11044.sm002 Jentsch et al. supplementary material 2 Jentsch et al. supplementary material

10.1192/bjo.2026.11044.sm003 Jentsch et al. supplementary material 3 Jentsch et al. supplementary material

10.1192/bjo.2026.11044.sm004 Jentsch et al. supplementary material 4 Jentsch et al. supplementary material

10.1192/bjo.2026.11044.sm005 Jentsch et al. supplementary material 5 Jentsch et al. supplementary material

10.1192/bjo.2026.11044.sm006 Jentsch et al. supplementary material 6 Jentsch et al. supplementary material

10.1192/bjo.2026.11044.sm007 Jentsch et al. supplementary material 7 Jentsch et al. supplementary material

10.1192/bjo.2026.11044.sm008 Jentsch et al. supplementary material 8 Jentsch et al. supplementary material

10.1192/bjo.2026.11044.sm009 Jentsch et al. supplementary material 9 Jentsch et al. supplementary material

## Data Availability

Because of privacy, the data that support the findings of this study are not publicly available. Data are available upon request from the corresponding author, M.C.J.

## Supplementary material

The supplementary material is available online at https://doi.org/10.1192/bjo.2026.11044

## Table 1 Long description

The table compares key study characteristics of three cohorts: MOTAR, PIDON/Vilnius, and tPEMF. It has 4 rows and 7 columns. The columns are labeled: Cohort, Total size, Participant status, Study design, Recruitment, MDD diagnosis, Inclusion criteria, and Exclusion criteria. Row 1: Cohort, MOTAR; Total size, 152; Participant status, 83 MDD, 69 Control; Study design, 16 weeks, two-arm treatment intervention study, partially randomised; Recruitment, Participants were recruited between 2012 and 2019 at a mental healthcare institution (GGZ Ingeest); MDD diagnosis, Participants were diagnosed at baseline using the DSM-IV and Composite International Diagnostic Interview. Inclusion criteria cases: having a current MDD diagnosis and aged 18-65 years. Assessments: MINI diagnostic interview, age 18-65 years, a HRSD-17 score equal to or higher than 11; Inclusion criteria, No lifetime MDD or anxiety disorder; Exclusion criteria, Antidepressant use in the past 2 weeks, current use of other psychotropic drugs (except for benzodiazepines), other clinically severe psychiatric disorders. Row 2: Cohort, PIDON/Vilnius; Total size, 84; Participant status, 27 MDD, 57 Control; Study design, Cross-sectional cohort study; Recruitment, Participants were recruited in collaboration with general practitioners, psychiatric care organisations and through advertisements in local and national newspapers. Participants were recruited from The Netherlands and Vilnius; MDD diagnosis, MINI diagnostic interview, age 18-65 years, a HRSD-17 score equal to or higher than 11; Inclusion criteria, No lifetime major depressive or anxiety disorder; Exclusion criteria, Pregnancy, presence of confounding psychiatric disorders. Row 3: Cohort, tPEMF; Total size, 50; Participant status, 50 MDD, 0 Control; Study design, Sham-controlled double-blind multicentre trial; Recruitment, Participants were recruited from major mental healthcare institutions from the northern part of The Netherlands, with MDD based on the DSM-IV criteria for MDD, who were in a first or recurrent depressive episode as assessed by the MINI; MDD diagnosis, HRSD-17; Inclusion criteria, Presence of at least a moderately severe depression (>17 on HRSD-17), non-responsiveness to one or more antidepressants given for at least 4 weeks during the current episode, age between 18 and 80 years; Exclusion criteria, MDD with psychotic features, other major psychiatric disorders, neurological disorders, suicidal thoughts, alcohol or drug misuse, changes in medication treatment scheme during the course of the study, use of benzodiazepines.

Navigate back to Table 1.
