# Supplementary material for: Feasibility of diagnosing major depressive disorder with a panel of serum and urine biomarkers
Source: BJPsych Open. 2026 Jun 15;12(4):e162. doi: 10.1192/bjo.2026.11044 (PMC13276772; doi:10.1192/bjo.2026.11044)
Supplement: Jentsch et al. supplementary material 1 — Jentsch et al. supplementary material [file S2056472426110448sup001.docx]

**S1:** List of biomarkers tested, matrix origin and Manufacturer.

|  | Matrix | Origin of Biomarker | Manufacturer |
| --- | --- | --- | --- |
| **1** | Acetyl-L-Carnitine | Serum | Abbexa |
| **2** | Acetyl-L-Carnitine | Urine | Abbexa |
| **3** | Aldosterone | Urine | R&D Systems |
| **4** | alpha1 anti-trypsin | Serum | Immundiagnostik |
| **5** | alpha1 anti-trypsin | Urine | Immundiagnostik |
| **6** | Apolipoprotein | Serum | R&D Systems |
| **7** | BDNF | Serum | R&D Systems |
| **8** | Free BDNF | Serum | R&D Systems |
| **9** | Total BDNF | Serum | R&D Systems |
| **10** | Calprotectin | Urine | Hycult Biotech |
| **11** | Calprotectin | Serum | Hycult Biotech |
| **12** | cAMP | Serum | R&D Systems |
| **13** | cGMP | Urine | R&D Systems |
| **14** | Cortisol | Urine | Diagnostics Biochem Canada Inc. |
| **15** | Cortisol | Serum | Diagnostics Biochem Canada Inc. |
| **16** | EGF | Serum | R&D Systems |
| **17** | EGF | Urine | R&D Systems |
| **18** | Endothelin-1 | Serum | R&D Systems |
| **19** | HVEM | Urine | R&D Systems |
| **20** | Isoprostane-2 | Urine | Northwest LLC |
| **21** | Leptin | Serum | R&D Systems |
| **22** | Lipocalin-2 | Urine | R&D Systems |
| **23** | LTB4 | Urine | R&D Systems |
| **24** | Midkine | Urine | CellMid |
| **25** | Myeloperoxidase | Serum | R&D Systems |
| **26** | Prolactin | Serum | Diagnostics Biochem Canada Inc. |
| **27** | Resistin | Serum | R&D Systems |
| **28** | Resistin | Urine | R&D Systems |
| **29** | Substance P | Serum | R&D Systems |
| **30** | Substance P | Urine | R&D Systems |
| **31** | Thromboxane | Serum | R&D Systems |
| **32** | Thromboxane | Urine | R&D Systems |
| **33** | TNFα receptor 2 | Serum | R&D Systems |
| **34** | Zonulin | Serum | R&D Systems |
