# Supplementary material for: Feasibility of diagnosing major depressive disorder with a panel of serum and urine biomarkers
Source: BJPsych Open. 2026 Jun 15;12(4):e162. doi: 10.1192/bjo.2026.11044 (PMC13276772; doi:10.1192/bjo.2026.11044)
Supplement: Jentsch et al. supplementary material 2 — Jentsch et al. supplementary material [file S2056472426110448sup002.docx]

**Supplementary 2: Method description RVI and results for each biomarker assay**

For some ELISAs we found large variance within the assay result of the QC samples indicating a low precision of the assay. Especially for biomarkers in which both the population variance and assay precision are low the diagnostic performance is poor since most variance in the samples will be caused by the assay and not by the biomarker distribution in the population. To evaluate this on an assay by assay basis an performance indicator, which we termed relative variability index (RVI), was developed. The variability of a sample (coefficient of variation, CV-value) relative to the full range of the sample concentrations provides a valuable indicator for the confidentiality of the outcome. It is not related to the measurement range of the assay itself. The RVI is defined as follows:

$$RVI=\frac{\mathrm{CV}all samples}{\mathrm{CV}\mathrm{QC}}$$

Where CV All samples Is the variability of all samples and CVQC-samples is the mean CV of the high and low QC samples. In our assays the CV All samples for each biomarker/body fluid combination was calculated from the measurement values of all participant samples in the 7 plates of one run. The CVQC was calculated from all 14 QC measurements (7 plates * 2 duplicates). Low variability and hence higher quality assurance is indicated with a high RVI. An arbitrary RVI value of 3.0 was considered as lowest acceptance criterion.

Results of the RVI for each biomarker are presented within the table below. 4 biomarkers did not meet the RVI criteria. Indicating a high assay variability and hence a lower quality assurance of results obtained

Results:

| **Biomarker** | **Matrix** |  | **Relative Variability Index (RVI)** |
| --- | --- | --- | --- |
| Creatinine | Urine |  | 10.5 |
| Lipocalin-2 | Urine |  | 11.9 |
| EGF | Serum |  | 9.6 |
| TNF RII | Serum |  | 5.3 |
| Calprotectin | Serum |  | 8.5 |
| Leptin | Serum |  | 12.5 |
| Endothelin-1 | Serum |  | 7.9 |
| Isoprostane-2 | Urine |  | 4.3 |
| Thromboxane | Serum |  | 8.6 |
| LTB4 | Urine |  | 8.3 |
| HVEM | Urine |  | 6.7 |
| cGMP | Urine |  | 4.4 |
| Cortisol | Urine |  | 7.1 |
| Cortisol | Serum |  | 5.2 |
| Calprotectin | Urine |  | 26.1 |
| Thromboxane | Urine |  | 4.8 |
| Midkine | Urine |  | 20.4 |
| cAMP | Serum |  | 3.3 |
| EGF | Urine |  | 9.3 |
| **Zonulin** | Serum |  | **2.7** |
| Aldosterone | Urine |  | 12.2 |
| **BDNF (BDNF free)** | Serum |  | **2.1** |
| BDNF (BDNF total) | Serum |  | 4.2 |
| Substance P | Serum |  | 4.7 |
| BDNF | Serum |  | 4.3 |
| Substance P | Urine |  | 7.3 |
| Apolipoprotein A1 | urine |  | 3.5 |
| Prolactin | serum |  | 9.4 |
| **Alpha1 antitrypsin** | serum |  | **2.1** |
| Alpha1 antitrypsin | urine |  | 72.6 |
| **Acetyl-L-Carnitine** | serum |  | **2.2** |
| Acetyl-L-Carnitine | urine |  | 3.5 |
| Myeloperoxidase | serum |  | 5.4 |
| Resistin | serum |  | 7.1 |
| Resistin | urine |  | 14.9 |
| **Red marked biomarkers did not pass the RVI criteria of >3.0** | | | |
