# Supplementary material for: Feasibility of diagnosing major depressive disorder with a panel of serum and urine biomarkers
Source: BJPsych Open. 2026 Jun 15;12(4):e162. doi: 10.1192/bjo.2026.11044 (PMC13276772; doi:10.1192/bjo.2026.11044)
Supplement: Jentsch et al. supplementary material 3 — Jentsch et al. supplementary material [file S2056472426110448sup003.docx]

**Supplementary 3:** Demographics total population

| Total population | | | | |
| --- | --- | --- | --- | --- |
| **# of samples** |  | Control |  | Case |
|  |  | 126 |  | 160 |
| **Gender** |  |  |  |  |
| Female |  | 66 |  | 95 |
| /Male |  | 60 |  | 65 |
| **Age** |  |  |  |  |
| Age |  | 47.2 |  | 43,6 |
| SD |  | 13.9 |  | 12.5 |
| **Ethnicity** |  |  |  |  |
| Caucasian |  | 116 |  | 141 |
| Other |  | 10 |  | 19 |
