# Supplementary material for: Feasibility of diagnosing major depressive disorder with a panel of serum and urine biomarkers
Source: BJPsych Open. 2026 Jun 15;12(4):e162. doi: 10.1192/bjo.2026.11044 (PMC13276772; doi:10.1192/bjo.2026.11044)
Supplement: Jentsch et al. supplementary material 4 — Jentsch et al. supplementary material [file S2056472426110448sup004.docx]

| **Table S4: QBP overview of validation training cohorts** | | | |  |  |  |  |  |
| --- | --- | --- | --- | --- | --- | --- | --- | --- |
|  |  |  |  |  |  |  |  |  |
| **Cohort** | **Tails** | **Limits** | **Biomarker Tested** | **Contributing biomarkers** | **AUC_real_** | **AUC_Random_** | **Significance** | **Permutations** |
| **Pidon + PEMF** | Actives only | 13/12 | 34 | 13 | 0.793 | 0.632 | P<0,0001 | 1290 |
| **Pidon + MOTAR** | Actives only | 15/12 | 34 | 13 | 0.770 | 0.590 | P<0,0001 | 1277 |
| **MOTAR + PEMF** | Actives only | 17/12 | 34 | 13 | 0.754 | 0.558 | P<0,0001 | 1527 |
