# Supplementary material for: Feasibility of diagnosing major depressive disorder with a panel of serum and urine biomarkers
Source: BJPsych Open. 2026 Jun 15;12(4):e162. doi: 10.1192/bjo.2026.11044 (PMC13276772; doi:10.1192/bjo.2026.11044)
Supplement: Jentsch et al. supplementary material 5 — Jentsch et al. supplementary material [file S2056472426110448sup005.docx]

Table S5: Active biomarkers within the respective (training) cohorts

| Total cohort |  |  | PEMF + MOTAR |  | Pidon + PEMF |  | Pidon + MOTAR |  |  |  |  |  |  |  |  |
| --- | --- | --- | --- | --- | --- | --- | --- | --- | --- | --- | --- | --- | --- | --- | --- |
|  |  |  |  |  |  |  |  |  |  |  |  |  |  |  |  |
| **Biomarkers** |  |  | **Biomarkers** |  | **Biomarkers** |  | **Biomarkers** |  |  |  |  |  |  |  |  |
| Acetyl-L-Carnitine_Serum |  |  | cAMP_Serum |  | Acetyl-L-Carnitine_Serum |  | BDNF_Serum |  |  | Active biomarkers within all cohorts | | | |  |  |
| BDNF_Serum |  |  | Cortisol_Serum |  | BDNF_Serum |  | cAMP_Serum |  |  | Active biomarkers within 3 cohorts | | | |  |  |
| cAMP_Serum |  |  | Endothelin_Serum |  | cAMP_Serum |  | Cortisol_Serum |  |  | Active biomarker within 2 cohorts | | | |  |  |
| Cortisol_Serum |  |  | Leptin_Serum |  | Endothelin-1_Serum |  | Endothelin-1_Serum |  |  | Active biomarkers within 1 cohort | | | |  |  |
| Endothelin-1_Serum |  |  | MPO_Serum |  | Leptin_Serum |  | Leptin_Serum |  |  |  |  |  |  |  |  |
| Leptin_Serum |  |  | Prolactin_Serum |  | Prolactin_Serum |  | Prolactin_Serum |  |  |  |  |  |  |  |  |
| Myeloperoxidase_Serum |  |  | TNFa receptor 2_Serum |  | Thromboxane_Serum |  | Thromboxane_Serum |  |  |  |  |  |  |  |  |
| Prolactin_Serum |  |  | Acetyl-L-Carnitine_CreatRatio |  | TNFa receptor 2_Serum |  | TNFa receptor 2_Serum |  |  |  |  |  |  |  |  |
| Thromboxane_Serum |  |  | HVEM_CreatRatio |  | Acetyl-L-Carnitine_CreatRatio |  | Acetyl-L-Carnitine_CreatRatio |  |  |  |  |  |  |  |  |
| TNFa receptor 2_Serum |  |  | Isoprostane-2_CreatRatio |  | Cortisol_CreatRatio |  | Cortisol_CreatRatio |  |  |  |  |  |  |  |  |
| Acetyl-L-Carnitine_CreatRatio |  |  | Resistin_CreatRatio |  | HVEM_CreatRatio |  | Isoprostane-2_CreatRatio |  |  |  |  |  |  |  |  |
| cGMP_CreatRatio |  |  | Substance P_CreatRatio |  | Resistin_CreatRatio |  | Substance P_CreatRatio |  |  |  |  |  |  |  |  |
| Cortisol_CreatRatio |  |  | Thromboxane_CreatRatio |  | Thromboxane_CreatRatio |  | Thromboxane_CreatRatio |  |  |  |  |  |  |  |  |
| HVEM_CreatRatio |  |  |  |  |  |  |  |  |  |  |  |  |  |  |  |
| Isoprostane-2_CreatRatio |  |  |  |  |  |  |  |  |  |  |  |  |  |  |  |
| LTB4_CreatRatio |  |  |  |  |  |  |  |  |  |  |  |  |  |  |  |
| Resistin_CreatRatio |  |  |  |  |  |  |  |  |  |  |  |  |  |  |  |
| Substance P_CreatRatio |  |  |  |  |  |  |  |  |  |  |  |  |  |  |  |
| Thromboxane_CreatRatio |  |  |  |  |  |  |  |  |  |  |  |  |  |  |  |
