# Supplementary material for: Feasibility of diagnosing major depressive disorder with a panel of serum and urine biomarkers
Source: BJPsych Open. 2026 Jun 15;12(4):e162. doi: 10.1192/bjo.2026.11044 (PMC13276772; doi:10.1192/bjo.2026.11044)
Supplement: Jentsch et al. supplementary material 6 — Jentsch et al. supplementary material [file S2056472426110448sup006.docx]

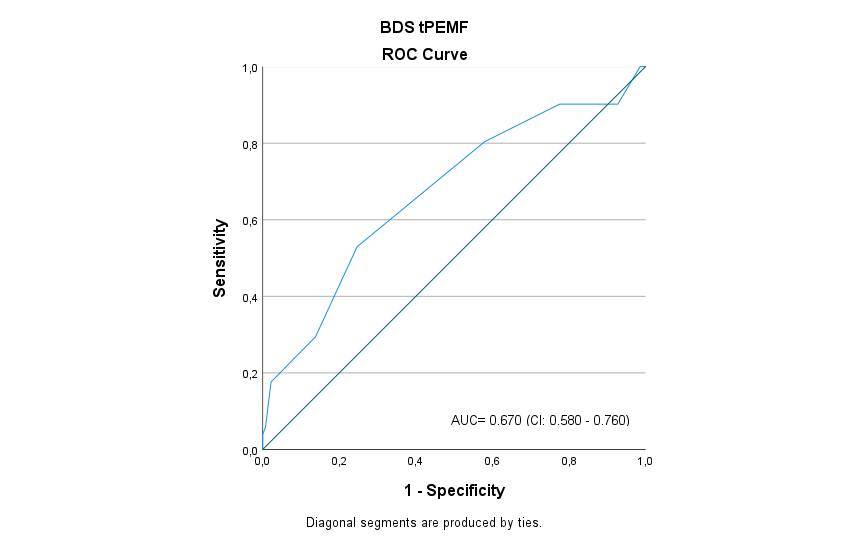


**Fig. S6a. ROC curve of BDS tPEMF.** ROC curve of BDS for the tPEMF cohort determined based on the internal-external validation.


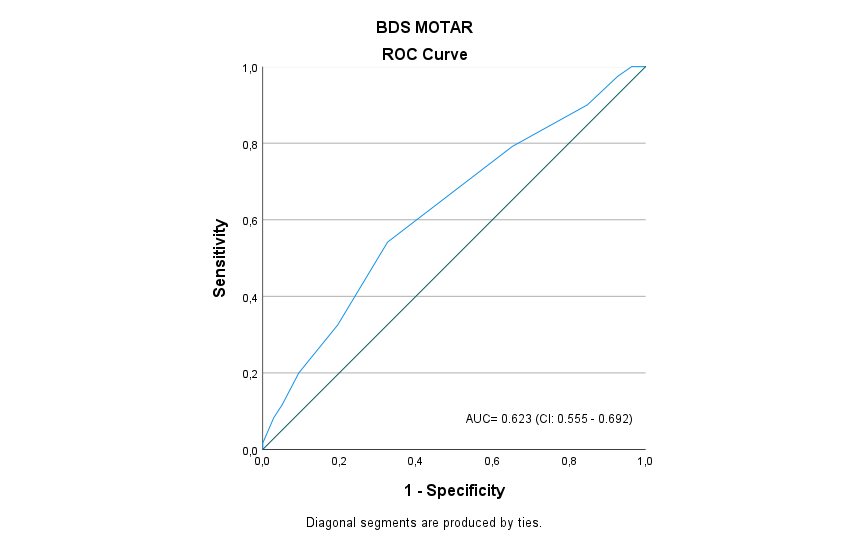


**Fig. S6b.ROC curve for BDS MOTAR**. ROC curve of BDS for the MOTAR cohort determined based on the internal-external validation.


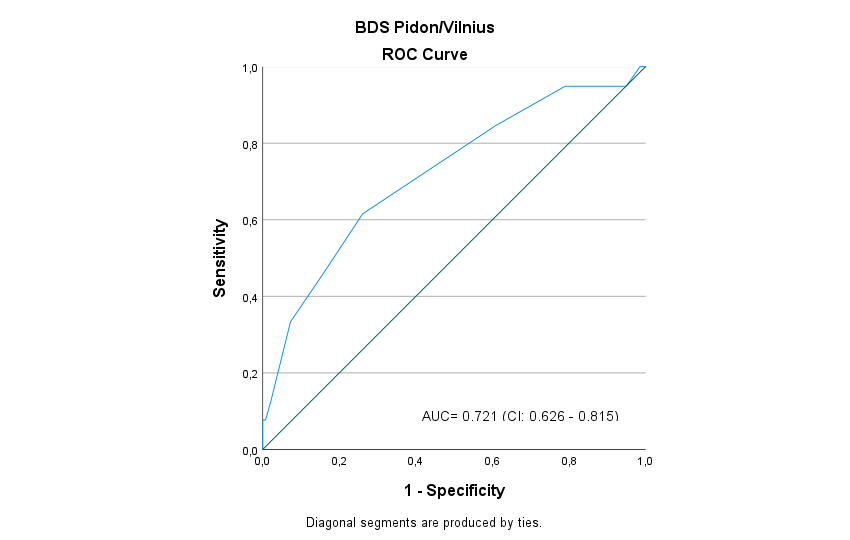


**Fig. S6c. ROC curve BDS Pidon/Vilnius.** ROC curve of BDS for the Pidon/Vilnius cohort determined based on the internal-external validation.
