# Supplementary material for: Feasibility of diagnosing major depressive disorder with a panel of serum and urine biomarkers
Source: BJPsych Open. 2026 Jun 15;12(4):e162. doi: 10.1192/bjo.2026.11044 (PMC13276772; doi:10.1192/bjo.2026.11044)
Supplement: Jentsch et al. supplementary material 7 — Jentsch et al. supplementary material [file S2056472426110448sup007.docx]

|  | BDS score (PEMF) | | | | | | |
| --- | --- | --- | --- | --- | --- | --- | --- |
| **Biomarkers** | -3 | -2 | -1 | 0 | 1 | 2 | 3 |
| BDNF_Serum | 13241,9 | 17558,65 | 22988,14 | 25238,14586 |  |  |  |
| cAMP_Serum | 108,727 | 83,1568 | 75,74959 | 43,72682413 | 39,57405 | 36,62439759 | 22,78520844 |
| Cortisol_Serum |  |  |  | 3,91684182 | 24,54374 | 26,683491 | 41,41867089 |
| Endothelin-1_Serum |  |  |  | 0,569928002 | 1,59742 | 1,856183446 | 2,27742925 |
| Leptin_Serum |  |  |  | 386,0923082 | 25752,36 | 37783,02497 | 47874,35584 |
| Prolactin_Serum |  |  |  | 2,403351473 | 17,05815 | 21,20233159 | 90,52913999 |
| Thromboxane_Serum |  |  |  | 184,5212014 | 98,54889 | 59,20244737 | 1,137423792 |
| TNFa receptor 2_Serum |  |  |  | 1422,016395 | 3472,022 | 3748,754397 | 3846,666867 |
| Acetyl-L-Carnitine_CreatRatio |  |  |  | 118,202462 | 1536,815 | 1815,435076 | 2869,493339 |
| Cortisol_CreatRatio | 4,348208 | 6,123699 | 31,36955 | 40,83564042 |  |  |  |
| Isoprostane-2_CreatRatio | 0,357539 | 0,376062 | 0,735377 | 0,926841584 |  |  |  |
| Substance P_CreatRatio | 531,6727 | 312,2966 | 259,1963 | 1,4 |  |  |  |
| Thromboxane_CreatRatio |  |  |  | 12,93764245 | 10,05256 | 8,822256216 | 5,886740531 |

**Tables S7: BDS scoring table for calculating the BDS score of each validation cohort based on concentration ranges**

|  | BDS score (Pidon/Vilnius) | | | | | | |
| --- | --- | --- | --- | --- | --- | --- | --- |
| **Biomarkers** | -3 | -2 | -1 | 0 | 1 | 2 | 3 |
| cAMP_Serum | 108,727 | 78,41259 | 71,70262 | 43,72682413 | 39,57405 | 36,62439759 | 20,01300527 |
| Cortisol_Serum |  |  |  | 3,574979946 |  | 26,683491 | 41,41867089 |
| Endothelin-1_Serum |  |  |  | 0,569928002 | 1,59742 | 1,856183446 | 2,27742925 |
| Leptin_Serum |  |  |  | 386,0923082 | 25752,36 | 37783,02497 | 47874,35584 |
| Myeloperoxidase_Serum | 20754,52 | 37609,68 | 44777,2 | 55448,48333 |  |  |  |
| Prolactin_Serum |  |  |  | 4,858692343 | 4,31449 | 3,31307857 | 2,403351473 |
| TNFa receptor 2_Serum |  |  |  | 1191,41247 | 3472,022 | 3748,754397 | 3846,666867 |
| Acetyl-L-Carnitine_CreatRatio |  |  |  | 118,202462 |  | 1815,435076 | 2869,493339 |
| HVEM_CreatRatio | 12918,48 | 18275,47 | 25541,6 | 35029,20346 |  |  |  |
| Isoprostane-2_CreatRatio | 0,497466 | 0,611467 | 0,754027 | 0,898452182 |  |  |  |
| Resistin_CreatRatio |  |  |  | 8,501653055 | 25815,91 | 33842,93697 | 76110,34021 |
| Substance P_CreatRatio | 555,8011 | 357,312 | 276,188 | 1,4 |  |  |  |
| Thromboxane_CreatRatio |  |  |  | 12,93764245 | 10,05256 | 8,822256216 | 5,498313711 |

|  | BDS score (MOTAR) | | | | | | |
| --- | --- | --- | --- | --- | --- | --- | --- |
| **Biomarkers** | -3 | -2 | -1 | 0 | 1 | 2 | 3 |
| Acetyl-L-Carnitine_Serum |  |  |  | 20,25128807 | 233,8501 | 264,7321345 | 345,3516258 |
| BDNF_Serum | 13241,9 | 17272,98 | 22709,92 | 26039,36864 |  |  |  |
| cAMP_Serum | 104,3491 | 80,05745 | 74,30401 | 20,01300527 |  |  |  |
| Endothelin-1_Serum |  |  |  | 0,705777258 | 1,59742 | 1,856183446 | 2,27742925 |
| Leptin_Serum |  |  |  | 613,0908764 | 25752,36 | 37783,02497 | 47874,35584 |
| Prolactin_Serum |  |  |  | 4,858692343 | 4,31449 | 3,31307857 | 2,946734328 |
| Thromboxane_Serum | 207,4646 | 95,00359 | 57,52234 | 4,09127341 | 2,759149 | 1,44287812 | 1,137423792 |
| TNFa receptor 2_Serum |  |  |  | 1191,41247 | 3472,022 | 3748,754397 | 3846,666867 |
| Acetyl-L-Carnitine_CreatRatio |  |  |  | 118,202462 | 1536,815 | 1815,435076 | 2869,493339 |
| Cortisol_CreatRatio |  |  |  | 31,47112047 | 22,73646 | 17,10974322 | 4,348208245 |
| HVEM_CreatRatio |  | 11221,74 | 25685,43 | 34040,71248 |  |  |  |
| Resistin_CreatRatio | 107,0301 | 841,8883 | 2376,541 | 3522,867098 | 25815,91 | 33842,93697 | 76110,34021 |
| Thromboxane_CreatRatio |  |  |  | 12,93764245 | 10,05256 | 8,822256216 | 5,498313711 |
