# Supplementary material for: Feasibility of diagnosing major depressive disorder with a panel of serum and urine biomarkers
Source: BJPsych Open. 2026 Jun 15;12(4):e162. doi: 10.1192/bjo.2026.11044 (PMC13276772; doi:10.1192/bjo.2026.11044)
Supplement: Jentsch et al. supplementary material 8 — Jentsch et al. supplementary material [file S2056472426110448sup008.docx]

**S8. BDS model performance characteristics for each sub cohort.**


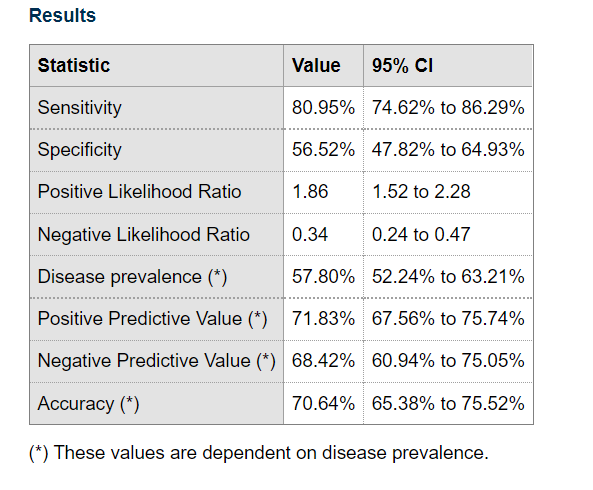


**Figure S8A BDS model performance characteristics for MOTAR.** BDS score criterium is set on > -0.5. the balanced accuracy rate is 0,69.


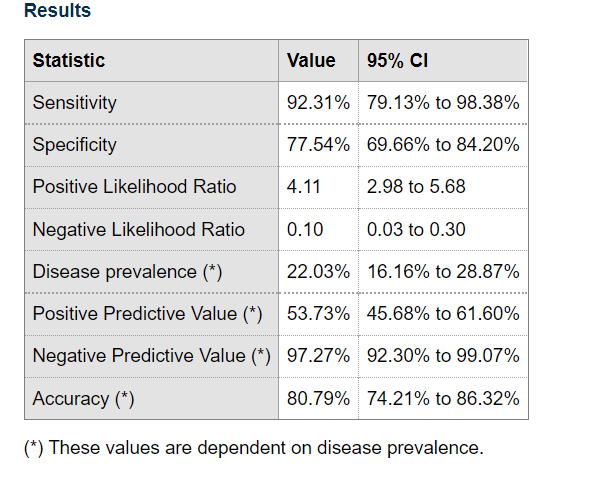


**Figure S8B. BDS model performance characteristics for tPEMF**. Bds score criterium is set on >1,5. The balanced accuracy of rate is 0.85


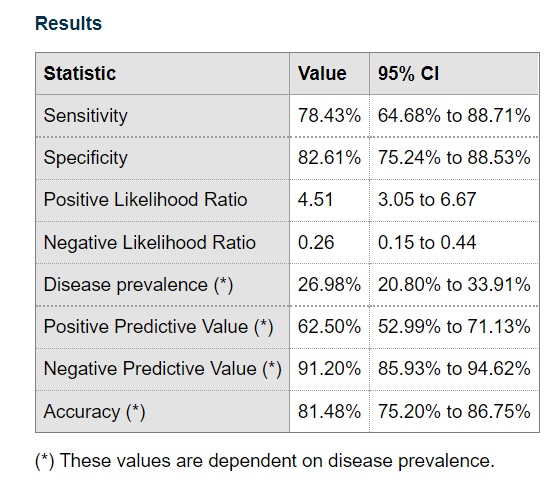


**Figure S8C. BDS model performance characteristics for Pidon/Vilnius**. BDS score criterium is set on >0.5. The balanced accuracy rate is 0,81.


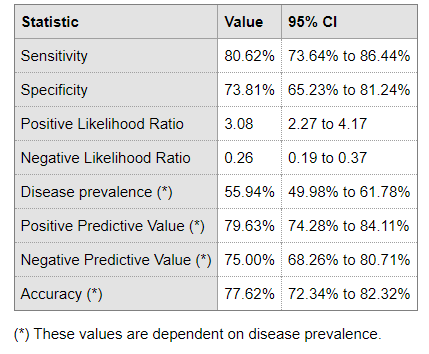


**Figure S8D. BDS model performance characteristics for total cohort.** BDS score criterium is set on >2.5. the balanced accuracy rate 0,77.
